# Comparison of *Helicobacter pylori* in hospitalized COVID‐19 patients with and without gastrointestinal symptoms

**DOI:** 10.1002/jgh3.70020

**Published:** 2024-09-21

**Authors:** Amin Saeedi, Afshin Mohammad Bagheri, Rasoul Raesi, Kiavash Hushmandi, Salman Daneshi, Asma Amiri Domari, Mohammadhossein Gholamzadeh, Shiva Kargar

**Affiliations:** ^1^ Department of Internal Medicine, School of Medicine, Imam Khomeini Hospital Jiroft University of Medical Sciences Jiroft Iran; ^2^ Department of Nursing Torbat Jam Faculty of Medical Sciences Torbat Jam Iran; ^3^ Department of Health Services Management School of Health, Mashhad University of Medical Sciences Mashhad Iran; ^4^ Nephrology and Urology Research Center, Clinical Sciences Institute Baqiyatallah University of Medical Sciences Tehran Iran; ^5^ Department of Public Health, School of Health Jiroft University of Medical Sciences Jiroft Iran; ^6^ Department of Surgery, School of Medicine Imam Khomeini Hospital Jiroft University of Medical Sciences Jiroft Iran; ^7^ School of Medicine Imam Khomeini Hospital Jiroft University of Medical Sciences Jiroft Iran; ^8^ Department of Epidemiology School of Health, Zahedan University of Medical Sciences Zahedan Iran

**Keywords:** COVID‐19, gastrointestinal, *Helicobacter pylori* (*H. pylori*), hospital, patient, symptom

## Abstract

**Background and Aim:**

*Helicobacter pylori* plays an important role in causing digestive diseases. The purpose of this study is to investigate *Helicobacter pylori* in COVID‐19 patients with and without gastrointestinal symptoms.

**Methods:**

In this case–control study, all patients with COVID‐19 admitted to Imam Khomeini Hospital in Jiroft city in 2021 were convenience sampled and divided into two homogeneous groups. Ninety‐five patients with COVID‐19, who presented with gastrointestinal symptoms, were included in the case group, while 95 patients with COVID‐19 without gastrointestinal symptoms were included in the control group. Noninvasive diagnostic methods, including serology and stool antigen tests, were used to identify *Helicobacter pylori* in the studied patients.

**Results:**

Fifty‐three people (55.8%) from the case group had *Helicobacter pylori*, and 48 (50.5%) from the control group had *Helicobacter pylori*. Among the 53 people from the case group, 27 (50.9%) were men and 26 (49.1%) were women. Nineteen people (35.8%) were taking pantoprazole, 10 people (18.8%) were taking nonsteroidal anti‐inflammatory drugs, 20 people (37.7%) were taking narcotics, and 7 people (13.2%) had peptic ulcer. Seven people (13.2%) had an H2 blocker, and 21 people had an underlying disease. A significant relationship between infection with *Helicobacter pylori* and the use of pantoprazole, nonsteroidal anti‐inflammatory drugs, narcotics, peptic ulcer, underlying disease, and H2 blocker in COVID‐19 patients with gastrointestinal symptoms and without gastrointestinal symptoms was present (*P*‐value < 0.05).

**Conclusion:**

The prevalence of *Helicobacter pylori* infection in patients with COVID‐19, who have gastrointestinal symptoms, is high and should be considered as a treatment criterion for people infected with COVID‐19.

## Introduction

In late December 2019, an unidentified pneumonia case was reported in Wuhan City, Hubei Province, China, whose clinical features were very similar to viral pneumonia. The World Health Organization (WHO) named this virus as COVID‐19 and the International Committee on Classification of Viruses as SARS‐COV‐2.^1^ This disease causes many different symptoms, with digestive problems being one of the most significant. Other organisms, such as *Helicobacter pylori*, can affect these symptoms. *Helicobacter pylori* is a type of Helicobacter bacterium and one of the most common microorganisms to infect humans globally.^2^

*Helicobacter pylori* is a spiral bacterium, but it can also change into a spherical shape. Both forms are viable and pathogenic, but the spherical form cannot be cultivated in a laboratory environment. *Helicobacter pylori* attaches to the stomach mucosa in both forms.^3^ *Helicobacter pylori* is found in the stomach and duodenum, and is related to gastroduodenal diseases. This bacterium is characterized by abundant production of urease enzyme, which is a virulence factor and can be used for diagnosis.^4^

*Helicobacter pylori* is the cause of most peptic ulcers and some gastrointestinal cancers, and it is the most important cause of stomach cancer and MALT lymphoma, but this microbe does not cause cancer in all individuals; about 15% of patients with long‐term infection may develop complications.^5^

According to estimates, about 25%–50% of people in developed countries and up to 70%–90% of people in developing countries are infected with this bacterium. In developing regions, up to 80% of the population may be infected with this infection by the age of 20, as the age increases, the probability of getting infected increases.^6^

Population density, living in unsanitary conditions, contaminated food or water, and contact with the stomach contents of infected people are known risk factors. Transmission of this disease occurs from person to person, and mainly through oral–oral or oral–fecal routes.^7^ According to unproven findings, some diets are effective in treating or reducing injuries caused by this disease.^8^

As mentioned, the gastrointestinal symptoms of coronavirus are wide‐ranging and can include anorexia, nausea, vomiting, diarrhea, and widespread abdominal pain. According to researchers, an increasing number of articles show that abdominal symptoms are a common manifestation of the coronavirus.^9^

A study conducted at the end of March in China shows that among 200 patients with coronavirus in three hospitals in Wuhan, China, about 50% reported at least one gastrointestinal complication.^10^ In another study, among 206 patients with coronavirus, 23% had only gastrointestinal symptoms, 43% had only respiratory symptoms, and 33% had both types of respiratory symptoms and gastrointestinal symptoms. The patients who had gastrointestinal symptoms ranged from diarrhea as their first sign.^11^

Patients with gastrointestinal symptoms sought medical care later than patients with respiratory symptoms, which was approximately 16 days compared with 11 days.^12^ New studies show that in patients with gastrointestinal symptoms, the time from the onset of symptoms to complete recovery from the virus is longer, and the virus may be seen in the feces of these patients to a greater extent than in patients with respiratory symptoms (73% vs. 14%).^13^

Many diseases, including those caused by *Helicobacter pylori*, result in digestive symptoms such as diarrhea, nausea, vomiting, and loss of appetite. Similarly, these symptoms can also occur in COVID‐19 patients. However, research on this topic has not been conducted in Iran or Jiroft City. Therefore, we decided to investigate the prevalence of *Helicobacter pylori* in COVID‐19 patients with and without gastrointestinal symptoms admitted to Imam Khomeini Hospital in Jiroft city.

## Method

### Study design

The present study is a retrospective (case–control) study. In this study, 190 COVID‐19 patients were selected and examined at Imam Khomeini Hospital (RA) in Jiroft city in 2021 using the convenience sampling method. The study participants were divided into two homogeneous groups. Homogenization was done based on variables such as age. The first group (case) of this study included patients with COVID‐19 with gastrointestinal symptoms, and the second group (control) of this study included patients with COVID‐19 without gastrointestinal symptoms. To identify *Helicobacter pylori* in the case and control groups, noninvasive diagnostic methods including serology and stool antigen tests were used. Stool samples of patients were exposed to kits containing polyclonal antibodies against *Helicobacter pylori* to find antibodies of bacterial genes. This pilot study was conducted on 95 patients with COVID‐19 with gastrointestinal symptoms and 95 patients with COVID‐19 without gastrointestinal symptoms. The data collection tool included a checklist made by the researcher, which recorded patients' demographic information (age, gender, smoking), gastrointestinal symptoms, underlying diseases (heart, diabetes, hypertension), use of pantoprazole, use of nonsteroidal anti‐inflammatory drugs, narcotic use, presence of a peptic ulcer, and H2 blocker.

### Statistical analysis

Using SPSS version 22 software, first the statistical indices related to the descriptive statistics of the frequency and percentage of the frequency were calculated. The Kolmogorov–Smirnov test was used to check the normality of the data, and then parametric tests such as the T‐test of two independent samples and Mann–Whitney, analysis of variance, Kruskal–Wallis, and Chi‐square test were used. The significance level in all statistical tests was considered less than 0.05.

### Ethical approval

The study was approved by the Research Ethics Committees of Jiroft University of Medical Sciences (Ethical code IR.JMU.REC.1400.059) and conducted in compliance with the relevant guidelines and regulations and the Declaration of Helsinki. Written informed consent was obtained from all patients to participate in this study. Patients were free to leave the study at any time. Also, the confidentiality of the patient information was ensured by the researcher.

## Results

In this study, 190 COVID‐19 cases were examined in Imam Khomeini Hospital in the city, and they were divided into two groups with gastrointestinal symptoms (case group) and without gastrointestinal symptoms (control group), each group having 95 patients. Fifty‐three people (55.8%) from the case group had *Helicobacter pylori* and 48 people (50.5%) from the control group had *Helicobacter pylori*. Statistically, there is a significant relationship between the prevalence of *Helicobacter pylori* in COVID‐19 patients with gastrointestinal symptoms (case group) and those without gastrointestinal symptoms (control group) (*P*‐value = 0.003).

Out of 53 people with digestive symptoms, 27 people (50.9%) were men and 26 people (49.1%) were women. Also, 2 people (3.7%) are under 20 years old, 10 people (18.9%) are 20–35 years old, 13 people (24.6%) are 35–50 years old, 27 people (50.9%) are 50 up to 80 years old, and 1 person (1.9%) was in the group over 80 years old. Out of 48 people with *Helicobacter pylori* without digestive symptoms, 8 people (17%) were in the age group of 20–35 years, 8 people (17%) were 35–50 years old, 26 people (55%) were 50–80 years old. Five people (11V%) were in the upper age group of 80 years. Statistically, there was a significant association with infected *Helicobacter pylori* in COVID‐19 patients with gastrointestinal symptoms (case group) and without gastrointestinal symptoms (control group) (*P*_value<0.05).

Out of 53 people with *Helicobacter pylori* with gastrointestinal symptoms, 19 people (35.8%) were taking pantoprazole, 10 people (18.8%) were taking nonsteroidal anti‐inflammatory drugs, 20 people (37.7%) were taking narcotics, and 7 people (13.2%) had peptic ulcer. Also, out of 53 people with *Helicobacter pylori* with gastrointestinal symptoms, 7 people (13.2%) had H2 blocker, 21 people had underlying diseases, 1 person (1.8%) had heart disease, 9 people (16.9%) had diabetes, and 11 people (20.7%) had hypertension. From a statistical point of view, there is a significant relationship between the prevalence of *Helicobacter pylori* and the use of pantoprazole, nonsteroidal anti‐inflammatory drugs, narcotics, peptic ulcer, underlying disease, and H2 blocker in COVID‐19 patients with gastrointestinal symptoms (case group) compared with those without gastrointestinal symptoms (control group) (*P*_value< 0.05). Table 1 shows the information related to the frequency of *Helicobacter pylori* and demographic variables in COVID‐19 patients in two groups of cases (with gastrointestinal symptoms) and controls (without gastrointestinal symptoms).

**Table 1:** Frequency of *Helicobacter pylori* and demographic variables in COVID‐19 patients with gastrointestinal symptoms (case group) and without gastrointestinal symptoms (control group)

Variables	Case group number (percent)	Control group number (percent)	*P*‐value
*Helicobacter pylori*			0.003
Yes	53 (55.8)	48 (50.5)	
No	42 (44.2)	47 (49.5)	
Age			0.04
<20	2 (3.7)	0	
20–35	10 (18.9)	8 (17)	
35–50	13 (24.6)	8 (17)	
50–80	27 (50.9)	26 (55)	
>80	1 (1.9)	5 (11)	
Sex			0.07
Women	27 (50.9)	26 (54.1)	
Men	26 (49.1)	22 (45.9)	
Taking pantoprazole			0.04
Yes	19 (35.8)	11 (22.9)	
No	34 (64.2)	37 (77.1)	
Taking nonsteroidal anti‐inflammatory drugs			0.03
Yes	10 (18.8)	6 (12.5)	
No	43 (81.2)	42 (87.5)	
Peptic ulcer			0.02
Yes	7 (13.2)	4 (8.3)	
No	46 (86.8)	44 (91.7)	
H2 blocker			0.001
Yes	7 (13.2)	5 (10.5)	
No	46 (86.8)	43 (89.5)	
Narcotic use			0.001
Yes	20 (37.7)	13 (27.1)	
No	33 (62.3)	35 (72.9)	
Underlying diseases			0.04
Heart disease	1 (1.8)	3 (6.2)	
Diabetes	9 (16.9)	8 (16.6)	
Hypertension	11 (20.7)	12 (25)	
No underlying disease	32 (60.6)	25 (52.2)	

## Discussion

Extra‐gastrointestinal diseases related to *Helicobacter pylori* infection have attracted the attention of many scientists around the world in recent years. *Helicobacter pylori* may be related to many intra‐ and extra‐gastrointestinal biological processes and is likely to determine and affect the occurrence of many extra‐gastrointestinal diseases.^14^ Therefore, this study was conducted to compare *Helicobacter pylori* in COVID‐19 patients with and without gastrointestinal symptoms admitted to Imam Khomeini Hospital, Jiroft City.

The prevalence of *Helicobacter pylori* in COVID‐19 patients was found to be 51.7%. In this regard, the study by Balamtekin et al., who investigated the effect of *Helicobacter pylori* on the clinical course of the COVID‐19 virus, showed that the prevalence of *Helicobacter pylori* in COVID‐19 patients is 28.7%,^15^ which is lower than the prevalence rate obtained in the present study. In interpreting the discrepancy in the prevalence of *Helicobacter pylori* in COVID‐19 patients in the present study compared with the study by Balamtekin et al., several points can be mentioned. These include the different demographic information of the studied patients, varying clinical conditions, different types and number of underlying diseases, and diverse geographical environments in the two studies, all of which can impact the prevalence of *Helicobacter pylori*.

In the study by Fallahi et al., the prevalence of *Helicobacter pylori* was 43.5%.^16^ During a study in Tehran, Rostaminejad et al. detected *Helicobacter pylori* in the biopsy samples of 91.3% of the studied subjects, which indicated the high prevalence of this bacterium in Tehran.^17^ In the city of Rotterdam, the Netherlands, the prevalence of *Helicobacter pylori* infection was found to be 22% by testing biopsy samples of patients who had been referred for routine endoscopy.^18^ In the study by Tanih et al., which was conducted on 254 dyspeptic patients in the Eastern Cape Province in South Africa, the prevalence of *Helicobacter pylori* infection was found to be 66.1%, although the prevalence of infection in Blacks was higher than the prevalence of infection in Whites.^19^ Nguyen et al., by testing the gastric biopsy samples of 279 patients undergoing endoscopy in Vietnam, reported the prevalence of this bacterial infection to be 65.6%.^20^ Another study is related to Japan, which estimated the prevalence of infection at 44.5%.^21^ During another study in Uruguay, the prevalence of *Helicobacter pylori* infection in patients of African origin was reported as 70%.^22^ Alazmi et al. conducted their study in Kuwait and reported the prevalence of *Helicobacter pylori* infection at 49.7%.^23^ In another study, the prevalence of this bacterial infection in Greek patients was 34%.^24^ As can be seen, the number of reported outbreaks is different, which can be due to the difference in crowding, environmental, geographical, social, economic, health, ethnic, and racial conditions of the studied populations.^19^

There is a significant relationship between the prevalence of *Helicobacter pylori* and the age of COVID‐19 patients, in other words, the prevalence of *Helicobacter pylori* in COVID‐19 patients increases with age. Since the beginning of the COVID‐19 pandemic, age has been recognized as a risk factor for morbidity and mortality. Although there have been changes in the researchers' observations over time, the incidence and mortality rates are still higher in the elderly, as according to the reports of the Centers for Disease Prevention and Control, a high percentage of deaths caused by the coronavirus occur in people over 65 years old. In America, the risk of infection is higher in the elderly. In confirmation of the results of the present study, the estimation of risk factors for contracting and dying from COVID‐19 was investigated by Caramelo et al. In China, the results of this study showed that age is a variable that is associated with a higher risk of contracting and dying from COVID‐19. It is associated with COVID‐19.^25^ In the study conducted by Grasselli et al., in the study of risk factors related to infection in patients with COVID‐19 in intensive care units in Italy, age was also introduced as a risk factor for the disease.^26^ In the study conducted in England, more than 90% of deaths were reported in people over 60 years of age, which is consistent with the global pattern and reports in this field.^27^ In the study by Fallah et al., there was a significant relationship between age and the prevalence of *Helicobacter pylori*, so the infection was more common in patients who were 40 years and more.^16^ It has been stated in various studies that the prevalence of this bacterium infection increases with age.^21^, ^28^, ^29^ This state is probably because, with increasing age, the frequency of contact with sources of infection increases and the chance of getting an infection increases.

The prevalence of *Helicobacter pylori* in COVID‐19 patients, whether with gastrointestinal symptoms (case group) or without gastrointestinal symptoms (control group), is higher in men than in women. Gender is also known as one of the risk factors that has been mentioned in most studies. So far, about 60% of the cases have been reported in men, which is consistent with the reports in this field regarding the increase in the death rate of men compared with women.^27^ In some research, it was reported that the reason for the increase in the incidence of COVID‐19 in men compared with women is more smoking and subsequently the increase in the presence of underlying diseases in men. According to Caramelo et al.'s study, in China, more deaths and infections are found in men, and this study is also consistent with other studies conducted in China and Italy.^25^, ^26^ In the study by WU et al. in the United States, the highest rate of infection was reported in elderly men with an underlying disease.^29^ Also, in the study by Sotoudehmanesh et al.^30^ and Malekzadeh et al.^31^ the majority of the population with *Helicobacter pylori* infection was men.

The results of this study showed that there is a statistically significant relationship between narcotic use and the prevalence of *Helicobacter pylori* infection in COVID‐19 patients. In some studies, it has been mentioned that narcotic use according to a systematic review and meta‐analysis published by the WHO, patients with a history of smoking had twice the risk of death compared with other patients.^32^ In the study by Fallah et al., a significant relationship between smoking and *Helicobacter pylori* infection was shown,^16^ in the study by Bastos et al.^33^ similar to the study by Fallah et al., a significant relationship between smoking and infection with this bacterium, but in several other studies, the lack of relationship between smoking and infection has been reported.^34^, ^35^

*Helicobacter pylori* infection can increase chronic inflammation with a decreased level of anti‐inflammatory factors such as IL‐6 and TNF‐a^36^; in addition, *Helicobacter pylori* has two variants ACE1 and ACE2 that express ACE2 in the effect of contact with the epithelial cells of the stomach increases and causes the regulation of the patient's immune system to be disturbed^37^; on the other hand, COVID‐19, like Sars, uses Angiotensin‐Converting Enzyme‐2 to enter the cell as a cell receptor.^38^ As a result of direct tissue damage caused by the virus, due to the presence of the ACE2 receptor and the presence of the viral nucleocapsid protein in the epithelial cells of the stomach, duodenum, rectum, and glandular enterocytes,^39^ as a result, *Helicobacter pylori* is a risk factor for the COVID‐19.

## Conclusion

In this study, the prevalence of *Helicobacter pylori* infection in patients with COVID‐19 was 51.7%; *Helicobacter pylori* infection in the examined COVID‐19 patients is considered a significant issue. The frequency of *Helicobacter pylori* infection in patients with COVID‐19 has gastrointestinal signs and also should be considered as treatment criteria for people infected with COVID‐19. However, more studies are needed to accurately prove the relationship between these types of infections and COVID‐19.

The recent investigation into the occurrence of *Helicobacter pylori* (*H. pylori*) infection among individuals diagnosed with COVID‐19 reveals an alarming rate of 51.7%. This finding highlights that *H. pylori* infection constitutes a substantial concern within the context of managing COVID‐19 cases. Notably, the presence of *H. pylori* in COVID‐19 patients often coincides with gastrointestinal symptoms, which may complicate diagnosis and treatment strategies.

Given the significance of this discovery, it becomes imperative for health policymakers to consider the following recommendations:

Incorporation of *H. pylori* testing: As part of routine diagnostic procedures for COVID‐19 patients presenting with gastrointestinal manifestations or those at high risk of *H. pylori* infection, such as older adults or those from regions where *H. pylori* prevalence is higher, healthcare providers must include *H. pylori* tests in their protocols. Early detection will enable timely intervention and potentially improve patient outcomes.

Collaborative efforts: To further investigate the potential link between *H. pylori* and COVID‐19, interdisciplinary collaboration between researchers specializing in infectious diseases, microbiology, and epidemiology would greatly benefit future endeavors. By pooling resources and expertise, we can better understand the complex interactions between these pathogens and develop targeted prevention and therapeutic measures.

Educational initiatives: Public awareness campaigns aimed at informing communities about the risks associated with *H. pylori* infection could help reduce its overall incidence. Additionally, educating medical professionals about the importance of screening for *H. pylori* in COVID‐19 patients will ensure appropriate care and management.

Research funding: Increased investment in scientific research focused on understanding the relationship between *H. pylori* and COVID‐19 is essential. With additional data, we can gain insights into the mechanisms underlying this association and identify effective preventive and treatment options.

While the current findings provide valuable information regarding the coexistence of *H. pylori* and COVID‐19, it is crucial to emphasize that more extensive investigations are required before definitively establishing causality between these conditions. Further exploration will undoubtedly contribute to our collective knowledge and guide evidence‐based decision‐making in public health policies.

## Consent for publication

We would like to express our sincere gratitude and appreciation to the respected Vice President of Research and Technology at Jiroft University of Medical Sciences, Imam Khomeini Hospital in Jiroft City, as well as all the people who helped us in carrying out this project.

## Data Availability

The data that support the findings of this study are available from the corresponding author upon reasonable request.
